# Bupropion Toxicity Causing Refractory Cardiogenic Shock Successfully Treated With Mechanical Circulatory Support: A Case Report

**DOI:** 10.7759/cureus.71137

**Published:** 2024-10-09

**Authors:** Salem Vilayet, Abubakr Adala, Munsef Barakat, Chakradhari Inampudi, George Carter, Aravind Menon

**Affiliations:** 1 Nephrology, Medical University of South Carolina, Charleston, USA; 2 Medicine, Leicester Royal Infirmary, Leicester, GBR; 3 Cardiology, Medical University of South Carolina, Charleston, USA; 4 Pulmonary and Critical Care Medicine, Medical University of South Carolina, Charleston, USA

**Keywords:** bupropion toxicity, cardiogenic shock, mechanical circulatory support, polymorphic ventricular tachycrdia, prolonged qtc interval

## Abstract

Bupropion is a norepinephrine-dopamine reuptake inhibitor that is commonly used as an antidepressant and for smoking cessation. Bupropion overdose can lead to serious side effects which include seizures, status epilepticus, and fatal arrhythmias. Managing bupropion toxicity is challenging as there is no effective antidote and treatment is largely supportive.

In this article, we present a case of bupropion toxicity causing profound cardiogenic shock which was treated successfully with mechanical circulatory support.

## Introduction

Bupropion is an antidepressant that acts via dopamine-norepinephrine reuptake inhibition. It has been increasingly prescribed for depressive disorders, smoking cessation, and attention deficit hyperactivity disorder (ADHD) [1]. Dosing of bupropion varies based on the specific indication and requires adjustment for patient renal function. Administering unadjusted doses in patients with renal impairment or end-stage renal disease can result in bupropion and its metabolites accumulating, which can lead to life-threatening toxicity [2]. In clinical practice, bupropion is available in both immediate-release and extended-release formulations. Side effects of bupropion can vary from mild (such as dry mouth, nausea, and insomnia) to severe. Life-threatening effects of bupropion toxicity include seizures and status epilepticus as well as critical cardiovascular dysfunction manifesting as QRS and QT interval prolongation, arrhythmias, and cardiogenic shock [1].

There is currently no known antidote for bupropion intoxication. The treatment options are limited to symptom management and providing supportive care. Studies have shown that activated charcoal may be used in cases of intoxication to partially inhibit absorption but should be administered within an hour of ingestion [3]. Hemodialysis is not recommended as the drug is mainly protein-bound and is highly lipophilic [3]. Lipid emulsion therapy may also be considered; however, the evidence for its use is inconsistent [3].

In this article, we present a case of cardiogenic shock secondary to bupropion toxicity refractory to medical management and eventually treated successfully by mechanical circulatory support (MCS) with veno-arterial extracorporeal membrane oxygenation (VA-ECMO) and Impella (Abiomed, Danvers, MA).

This case was presented as a poster at the 2023 CHEST Annual Meeting [4].

## Case presentation

A 49-year-old female was brought to the emergency room after being found confused in her room. Further investigation revealed an apparent suicide attempt with intentional co-ingestion of alcohol and an entire bottle of bupropion 300 mg XL tablets, the number of ingested pills was unknown. On initial assessment, she had a heart rate of 138 bpm, blood pressure of 165/100 mmHg, and normal oxygen saturation on room air. Her Glasgow Coma Scale (GCS) on arrival was 9. Shortly after arrival at the hospital, the patient developed tonic-clonic seizures. She was initially treated with lorazepam and levetiracetam and was subsequently intubated for airway protection and started on propofol and midazolam infusions. She was admitted to the intensive care unit (ICU) where she underwent continuous electroencephalogram (EEG) monitoring. CT brain was unremarkable, and initial laboratory findings are reported in Table 1.

**Table 1:** Initial laboratory findings PCO2: partial pressure of carbon dioxide, PH: potential hydrogen

Labs	Value	Normal range
Sodium	146 mmol/L	135-145 mmol/L
Potassium	3.6 mmol/L	3.5-5 mmol/L
Bicarbonate	16 mmol/L	22-29 mmol/L
Chloride	114 mmol/L	98-108 mmol/L
Glucose	106 mg/dL	70-100-mg/dL
Blood urea nitrogen (BUN)	11 mg/dL	7-11 mg/dL
Creatinine	0.6 mg/dL	0.6-1.1 mg/dL
Calcium	8.7 mg/dL	8.4-10.3 mg/dL
Alanine aminotransferase (ALT)	24 U/L	5-45 U/L
Aspartate aminotransferase (AST)	25 U/L	5-34 U/L
Bilirubin	0.2 mg/dL	0.2-1.2 mg/dL
Lactate	12 mmol/L	0.5-1.6 mmol/L
Albumin	4 g/dL	3.5-5 g/dL
Blood gases		
PH	7.21	7.35-7.45
PCO2	55 mmHg	35-45 mmHg
Bicarbonate	22 mmol/L	22-26 mmol/L
Toxicology screen		
Serum ethanol	245.3 mg/dL	<10mg/dL
Bupropion	570 ng/mL	
Hydroxybupropion	3800 ng/mL	
Salicylate	Negative	
Tricyclic antidepressants	Negative	
Acetaminophen	Negative	

**Figure 1:**
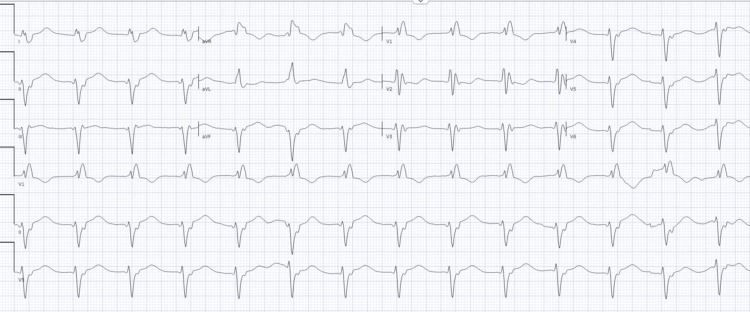
ECG showing right bundle branch block, idioventricular rhythm and prolonged QTc interval QTc: corrected QT

Within a few hours of her arrival, the patient went into shock with significant hypotension, with blood pressure of 80/50 mmHg and evidence of hypoperfusion, including cold peripheries and increasing serum lactate. Her electrocardiogram (ECG) revealed a junctional rhythm, prolonged corrected QT (QTc) interval (QTc = 606 ms), and a widened QRS complex with right bundle branch block (RBBB) morphology (Figure 1).

Her arrhythmia and conduction delay progressed to polymorphic ventricular tachycardia necessitating cardioversion and the administration of intravenous calcium gluconate, magnesium, lidocaine, and amiodarone. She was started on high-dose isotonic sodium bicarbonate infusion at 200 mL/hr and received two doses of lipid emulsion therapy. Transthoracic echocardiogram (TTE) on arrival showed a normal left ventricular ejection fraction (LVEF) of 55%; repeat TTE after less than 18 hours in the ICU revealed an LVEF of 20%.

Despite maximal inotropic and vasopressor support alongside adequate initial fluid resuscitation, the patient experienced progressive cardiogenic shock which was attributed to profound electromechanical cardiac dysfunction secondary to bupropion toxicity. In the setting of cardiogenic shock refractory to medical management, the patient was referred for mechanical circulatory support (MCS) as a bridge to recovery. Less than 24 hours after admission, she was cannulated for veno-arterial extracorporeal membrane oxygenation (VA-ECMO), and an Impella pump was placed simultaneously to decompress the left ventricle. Supportive care was continued in the ICU and the patient experienced a complete recovery, as evidenced by a repeat TTE showing an ejection fraction of 60% after 72 hours on MCS. Subsequently, the patient was successfully decannulated from VA-ECMO after six days and the Impella device (Abiomed, Danvers, MA) was removed after seven days; repeat echo off MCS confirmed cardiac recovery with a normal LVEF. She was successfully extubated after eight days and was discharged home 23 days after her initial presentation.

## Discussion

Bupropion toxicity cases have been increasing in the United States in recent years. America’s Poison Centers reported more than 18,000 cases of bupropion overdose, with 9,708 of these cases attributed to single exposures, leading to major adverse events in more than 600 patients and causing 18 fatalities [5].

Most cases of bupropion toxicity occur with a higher dose of extended-release formulation. Total doses exceeding 5 g carry a high risk for severe neurological and cardiovascular toxicity, which are the main causes of mortality in bupropion overdose [6]. After oral ingestion, bupropion is readily absorbed, with peak serum concentration occurring within two to five hours depending on the formulation used. It is extensively bound to proteins with about 84% bound to plasma proteins, and it has a large volume of distribution, around 19 L/kg at steady state [7]. Bupropion is metabolized by CYP2B6 to its active metabolite hydroxybupropion. Bupropion and its metabolites are excreted via urine (88%) and via feces (10%); less than 1% of bupropion is excreted unchanged in the urine [7]. The total elimination half-life of bupropion and its metabolites ranges from 20-37 hours [7]. 

Typical signs and symptoms of bupropion overdose are related to excessive adrenergic stimulation including xerostomia, irritability, insomnia, and weight loss; larger doses can also result in high blood pressure and mydriasis. Seizures, arrhythmias, and cardiogenic shock occur in severe toxicity [8].

Interestingly, the arrhythmogenic effects of bupropion may have a distinct mechanism as compared to other medications and toxins that lead to QRS widening and QTc prolongation. The effect of bupropion on the myocyte’s delayed rectifier potassium (Kir) channel is mild and concentration-dependent; thus with a normal therapeutic dose only mild QTc prolongation is seen, but in cases of bupropion overdose clinically significant QTc prolongation becomes evident [9]. It has also been suggested that bupropion-induced QRS widening is not related to the blocking of myocyte sodium channels but is rather due to the disruption of intercellular myocyte signaling by inhibiting myocardial gap junctions [9], which could explain the relative inefficacy of sodium bicarbonate infusion in treating bupropion-induced arrhythmia. Data regarding the utility of intravenous lipid emulsion therapy in bupropion toxicity are lacking but cases of its use as a salvage therapy have been reported [10].

Cardiogenic shock is characterized by inadequate cardiac output and tissue perfusion causing tissue hypoxia [11]. The most common causes of cardiogenic shock are advanced heart failure and acute myocardial infarction [8]. The treatment, clinical course, and outcomes vary depending on the cause. Inotropes are employed as an initial therapeutic approach for over 90% of patients admitted to the ICU with cardiogenic shock, and vasopressors are used to treat concomitant vasodilation in the systemic circulation [8]. In patients with combined decreased cardiac output and decreased systemic vascular resistance, MCS may be necessary to provide adequate tissue perfusion and to maintain acceptable blood pressure [12].

Toxicity-related cardiogenic shock can cause refractory hypotension and profound tissue hypoxia. Vasopressors are commonly used in toxicity-related cardiogenic shock with the goal of sustaining adequate mean arterial pressure; however, their efficacy has been questioned in the setting of animal studies demonstrating an increase in cardiac demand ischemia and perfusion mismatch [13].

## Conclusions

Bupropion overdose may result in cardiovascular toxicity, which in turn may lead to arrhythmia and cardiogenic shock. These life-threatening conditions can prove challenging to treat using conventional medical interventions due to the particular mechanisms by which bupropion toxicity affects myocytes and the lack of effective antidotes or drug-clearing treatments.

In our case, cardiogenic shock was induced by bupropion overdose resulting in myocardial toxicity with conduction delays progressing to critical arrhythmia and depressed ventricular function accompanied by profound systemic vasodilation. This shock was unresponsive to initial therapy including inotropes and vasopressors but was successfully treated with transition to MCS via VA-ECMO and Impella pump. The patient made a full recovery and was successfully weaned from all life support, suggesting that early utilization of MCS for treatment-refractory cardiogenic shock may be beneficial in cases of bupropion toxicity.
